# Osteoclast profile of medication-related osteonecrosis of the jaw secondary to bisphosphonate therapy: a comparison with osteoradionecrosis and osteomyelitis

**DOI:** 10.1186/s12967-017-1230-8

**Published:** 2017-06-06

**Authors:** Christian Gross, Manuel Weber, Kay Creutzburg, Patrick Möbius, Raimund Preidl, Kerstin Amann, Falk Wehrhan

**Affiliations:** 1 Department of Oral and Maxillofacial Surgery, University Hospital, Friedrich-Alexander-University Erlangen-Nürnberg (FAU), Erlangen, Germany; 2 Department of Nephropathology, Institute of Pathology, Friedrich-Alexander-University Erlangen-Nürnberg (FAU), Erlangen, Germany; 3 Department of Oral and Maxillofacial Surgery, Research Laboratory, Friedrich-Alexander-University Erlangen-Nürnberg (FAU), Glückstrasse 11, 91054 Erlangen, Germany

**Keywords:** Bisphosphonate, Osteonecrosis, Osteomyelitis, Osteoradionecrosis, Osteoclasts, TRAP, DC-STAMP, MRONJ, BRONJ

## Abstract

**Background:**

The medication-related osteonecrosis of the jaw secondary to bisphosphonate therapy [MRONJ (BP)] is characterized by non-healing exposed bone in the maxillofacial region. The pathogenesis of MRONJ (BP) is not fully understood. Giant, hypernucleated, inactive osteoclasts were found in MRONJ (BP) tissues, which indicated that accelerated cell–cell fusion might play a role. Dendritic cell-specific transmembrane protein (DC-STAMP) is associated with the cell–cell fusion of osteoclasts and precursor cells. Tartrate-resistant acid phosphatase (TRAP) is essential for osteoclastic bone resorption. The cell–cell fusion, as part of the osteoclastogenesis, and the resorptive activity can determine the morphology of osteoclasts. This study analyzed jaw bone from patients with MRONJ (BP), osteomyelitis (OM) and osteoradionecrosis (ORN) because a comparison with the osteoclast profiles of OM and ORN is essential for characterizing the osteoclast profile of MRONJ (BP).

**Methods:**

Formalin-fixed routine jaw bone specimens from 70 patients [MRONJ (BP) n = 30; OM: n = 15, ORN: n = 15, control: n = 10] were analyzed retrospectively for osteoclast quantity, morphology and the expression of TRAP and DC-STAMP. The specimens were processed for hematoxylin and eosin staining (H&E), histochemistry (TRAP) and immunohistochemistry (anti-DC-STAMP) and were analyzed via virtual microscopy.

**Results:**

The quantity, diameter and nuclearity of osteoclasts were significantly higher in MRONJ (BP) specimens than in OM, ORN and control specimens. Giant, hypernucleated osteoclasts were detected in MRONJ (BP) specimens only. Osteoclastic TRAP expression was lower in MRONJ (BP) and ORN specimens than in OM and control specimens. The DC-STAMP expression of osteoclasts and mononuclear cells was significantly higher in MRONJ (BP) and ORN specimens than in OM and control specimens.

**Conclusions:**

This study indicates that the osteoclast profile of MRONJ (BP) is characterized by osteoclast inactivation and a high cell–cell fusion rate; however, the presence of giant, hypernucleated osteoclasts cannot be attributed to increased DC-STAMP-triggered cell–cell fusion alone. The incidental characterization of the osteoclast profiles of OM and ORN revealed differences that might facilitate the histopathological differentiation of these diseases from MRONJ (BP), which is essential because their therapies are somewhat different.

## Background

Since nitrogenous bisphosphonates have become part of the therapeutic approach to osteoporosis, M. Paget, multiple myeloma, and osseous metastases of solid tumors, many case reports and studies about severe osteonecrosis of the jaw, which is associated with the application of these antiresorptive drugs, have been published [1–7]. The growing epidemic of this BP-induced osteonecrosis of the jaw (ONJ) was first described by Marx in 2003 and is currently considered the most common type of osteonecrosis of the human jaw [8, 9]. Because there is evidence that newer antiresorptive drugs (e.g., Denosumab) could also cause ONJ, the International Task Force on Osteonecrosis of the Jaw currently recommends using the term medication-related osteonecrosis of the jaw (MRONJ) [10]. The current definition of MRONJ is restricted to clinical and anamnestic parameters. MRONJ is defined as (1) exposed bone in the maxillofacial region that does not heal within 8 weeks after identification by a health care provider; (2) exposure to an antiresorptive agent; and (3) no history of radiation therapy to the craniofacial region [10]. MRONJ secondary to bisphosphonate therapy [MRONJ (BP)] needs to be highlighted because bisphosphonates still play the dominant role within the group of antiresorptive drugs. The prevalence of this side effect of bisphosphonate therapy is high and reaches approximately from 0.1 to 19%, depending on the underlying disease, the treatment period, and further risk factors [3–7]. Studies analyzing the histological properties of jaw bone specimens from MRONJ (BP) patients exist; however, these studies show somewhat contradictory results [11, 12].

Osteomyelitis (OM) is defined as an inflammatory condition of bone, which begins as an infection of the medullary cavity, rapidly involves the haversian systems, and extends to involve the periosteum of the affected area [13]. The inflammation of bone tissue is associated with bone resorption, destruction and dysfunction. The most common cause of OM of the jaw is a polymicrobial odontogenic infection. Usually adult males are affected. OM of the jaw more commonly occurs in the mandible than in the maxilla [13, 14]. Depending on the clinical course, it is commonly further distinguished between acute, subacute and chronic osteomyelitis [15, 16].

Osteoradionecrosis (ORN) is defined as exposed devitalized irradiated bone that does not heal over a period of 3–6 months, a positive anamnesis for irradiation, and the absence of local neoplastic disease [17, 18]. It is associated with a high morbidity and recurrence risk. An potential incurability has been discussed [19]. The risk of developing ORN is increased in patients with poor oral health, because traumatic dental events (especially extractions) increase the risk of onset of ONJ [18]. Furthermore, the risk for ORN is positively related to radiation doses of > 60 Gy [20].

Giant, hypernucleated and inactive osteoclasts were observed in samples from patients after bisphosphonate therapy and from MRONJ (BP) patients [21, 22]. The currently postulated effects of nitrogenous bisphosphonates on osteoclasts, namely, the disturbance of the mevalonate metabolism, hardly explain these findings [23]. It is assumed that high concentrations of calcium in the extracellular space, which are generated during osteoclastic bone resorption, induce the apoptosis of osteoclasts [24, 25]. From this assumption, Weinstein et al. concluded that the emergence of giant, hypernucleated osteoclasts in MRONJ (BP) is caused by the bisphosphonate-mediated inhibition of osteoclastic bone resorption that prevents osteoclast apoptosis [21]. However, the inhibition of apoptosis could not explain the increase in size and nuclearity directly because these processes of osteoclasts maturation usually require cell–cell fusion [26]. Thus, evaluating the role of cell–cell fusion in the genesis of these abnormal osteoclasts is essential.

Dendritic cell-specific transmembrane protein (DC-STAMP), a dimeric membrane-bound protein, depends on RANK–RANKL-signaling and has been determined to be essential for the cell–cell fusion of mononuclear osteoclasts in murine settings and in experiments with human cells [27–33]. This protein has been shown to be expressed in dendritic cells and macrophages but predominantly in osteoclasts [34]. RNA interference studies on human osteoclasts demonstrated an essential role for osteoclastic cell–cell fusion and resorptive activity [28]. DC-STAMP-deficient mice did not develop multinucleation of osteoclasts [35].

Tartrate-resistant acid phosphatase (TRAP), a metalloenzyme, has been used as a histochemical marker for osteoclasts in the past several years, although it is also present in macrophages, dendritic cells and several other cell types [36, 37]. In vitro, a positive correlation between osteoclastic TRAP-secretion and bone resorption was described [38]. Furthermore, the serum TRAP-5b concentration was shown to correlate with the rate of bone resorption [39] in vivo. TRAP-deficient mice showed increased bone density [40].

The status of DC-STAMP and TRAP might be critical for understanding the presence of altered osteoclasts in MRONJ (BP) tissues. Moreover, in combination with the analysis of osteoclast quantity and morphology, these proteins could contribute to a characterization of the osteoclast profile of MRONJ (BP). This study analyzed jaw bone samples from patients with MRONJ (BP), OM and ORN because a comparison with the osteoclast profiles of OM and ORN is essential for characterizing the osteoclast profile of MRONJ (BP). Comparisons regarding the histology of these pathological conditions, which all feature clinical similarities and cause destruction of the jaw bone, do exist. However, attempts to characterize osteoclasts histologically in these conditions have shown no consistent results to date [41–44].

The aims of this study were as follows: (1) to analyze and compare osteoclasts in terms of the quantity, morphology, and expression of TRAP and DC-STAMP in formalin-fixed routine jaw bone specimens from patients with MRONJ (BP), OM, ORN and normal jaw bone (control); (2) to investigate the status quo of osteoclast activity and cell–cell fusion (mononuclear cells and osteoclasts) in MRONJ (BP) specimens; and (3) to investigate if disease-characteristic osteoclast profiles exist that might facilitate the histopathological differentiation of MRONJ (BP), OM and ORN.

## Methods

### Patient selection and specimen harvesting

For the present study, routine jaw bone specimens (n = 70) from patients (n = 70), treated in the Department of Oral and Maxillofacial Surgery of the University Hospital Erlangen between 2007 and 2015, were analyzed retrospectively [MRONJ (BP): n = 30, ORN: n = 15, OM: n = 15]. Specimens from histopathologically inconspicuous jaw bones (10 patients) were used as controls. The analyzed specimens were collected intraoperatively as part of routine histopathological diagnostics. Bone specimens were fixed in 4% formalin immediately after extraction. Diagnoses were confirmed by the Department of Pathology of the University Hospital Erlangen. The presence of clinical criteria during the time of extraction were verified by the review of medical records and radiographs (depending on the specific clinical criteria).

Beyond histopathological confirmation, the clinical criteria for the selection of MRONJ (BP) specimens were as follows: (I) evidence of more than 8 weeks of exposed jaw bone; (II) documented therapy with bisphosphonates; and (III) no radiotherapy. Patients with a positive medication history for denosumab, bevacizumab, pazopanib, sunitinib, mTOR inhibitors and sorafenib were not included in this group.

Beyond histopathological confirmation, the clinical criteria for the selection of ORN specimens were as follows: (I) evidence of devitalized and exposed jaw bone in a previously irradiated field in the absence of local neoplastic processes and (II) no therapy with bisphosphonates.

Beyond histopathological confirmation, the clinical criteria used to select OM specimens were as follows: (I) evidence of chronic inflammatory processes in the jaw bone; (II) no therapy with bisphosphonates; and (III) no radiotherapy. Patients with a diagnosis of primary chronic OM (non-bacterial cause) were not included in this study.

The control specimens originated from patients who (I) had never been treated with bisphosphonates or local radiation; (II) were not taking medications significantly affecting jaw bone homeostasis; and (III) did not suffer from intraoral inflammation; (IV) periodontitis; (V) neoplastic malignancies or (VI) relevant systemic diseases (e.g., osteoporosis) at the time of extraction.

The presence of neoplastic malignancies near the extraction site on the extraction date disqualified the patients from selection for this study. For detailed patient data, see Table 1.

**Table 1:** Patient data

	MRONJ (BP)	OM	ORN	CONTROL
Number of patients	30	15	15	10
Sex	53.3% women (16)	53.3% women (8)	13.3% women (2)	40% women (4)
Age (years)	Ø 67.8 ± 8.89	Ø 43.6 ± 25.20	Ø 57 ± 7.89	Ø 33.8 ± 16.17
(Primary) diagnosis	33.33% prostate cancer (10), 30% breast cancer (9), 20% multiple myeloma (6),10% osteoporosis (3), 0.33% renal cell carcinoma (1), 0.33% vertebral sclerosis (1)	86.6% chronic osteomyelitis (13), 13.3% acute osteomyelitis (2)	60% SCC oral cavity (9), 13.3% SCC oropharynx (2), 6.6% SCC hypopharynx (1), 6.6% SCC tonsil (1), 6.6% SCC cranial skin (1), 6.6% CUP	50% facial fracture (5), 20% dysgnathia (2), 10% cleft lip and palate (1), 10% wisdom tooth extraction (1), 10% arch ratio anomaly (1)
Extraction location	76.7% lower jaw (23), 23.3% upper jaw (7)	100% lower jaw (15)	100% lower jaw (15)	80% lower jaw (8), 20% upper jaw (2)
Additional information	100% nitrogenous. BPs (30): 70% zoledronate (21), 13.3% alendronate (4), 6.6% risedronate (2), 6.6% ibandronate (2), 3.3% pamidronate (1)		Ø total reference dose in the mandibular region: 68 Gy (The applicated dose was set individually by the radiotherapists)	

Age is shown as the mean and standard deviation

Ø, average; min, minimum; max, maximum; BP, bisphosphonate; MRONJ (BP), medication-related osteonecrosis of the jaw secondary to bisphosphonate therapy; OM, osteomyelitis; ORN, osteoradionecrosis; SCC, squamous cell carcinoma; CUP, cancer of unknown primary

### Light microscopy, histochemistry and immunohistochemistry (IHC)

The formalin-fixed samples were decalcified and embedded in paraffin before being sliced in sections of 3-μm thickness using a microtome (RM2165, Leica, Nussloch, Germany). Special microscope slides with improved adhesion were used (SUPERFROST ULTRA PLUS, Gerhard Menzel GmbH, Braunschweig, Germany). The primary preparation for staining included dewaxing in xylene and rehydration in graded propanol and finally in distilled water.

### Hematoxylin and eosin staining (H&E) was carried out according to standard protocols

TRAP-staining was performed using a TRAP-detection system (TRACP & ALP double-stain Kit, MK 300, Takara Bio, Kusatsu, Japan; preparation of reagents following instructions, TRAP detection only), that generates an azoic dye (purplish-red color) if the enzyme is present. Sections, covered with prepared working solution, were incubated at 37 °C in a wet chamber for 5 h. Nuclear counterstaining with hematoxylin (CS700, Dako Deutschland GmbH, Hamburg, Germany) followed.

Immunohistochemical staining was performed using a polymeric method and an automated staining device (Autostainer plus, DakoCytomation, Dako Deutschland GmbH, Hamburg, Germany). An EnVision Detection System Peroxidase/diaminobenzidine (DAB), Rabbit/Mouse (K5007 HRP/DAB+, Dako Deutschland GmbH, Hamburg, Germany) was used as the staining kit. Antigen retrieval consisted of specimen treatment with ethylenediaminetetraacetic acid (EDTA) (dilution 1:100, PMB4-125, Antigen Retrieval Buffer 4, Spring Bioscience, CA, USA) at 66.7 °C for 5 h. To reduce background staining artifacts, peroxidase- and protein-blocking were performed prior to protein detection as follows: (1) peroxidase-blocking for 5 min. (S2023, DAKO REAL, Peroxidase-Blocking Solution, Dako Deutschland GmbH, Hamburg, Germany); (2) protein-blocking for 10 min. (X0909, Protein Block Serum-Free, Dako Deutschland GmbH, Hamburg, Germany). The target proteins were detected by incubating tissues with an anti-DC-STAMP-antibody (dilution 1:25; incubation time: 35 min HPA062520, Anti-DC-STAMP, rabbit, polyclonal, Atlas Antibodies AB, Stockholm, Sweden). Further processing with conjugated dextran (25 min) and DAB+ chromogen (5 min) visualized antibody-marked proteins. Nuclear counterstaining with hematoxylin (CS700, Dako Deutschland GmbH, Hamburg, Germany) followed. Positive and negative controls were included in each staining series.

### Cell morphology and quantitative analysis

**Fig. 1:**
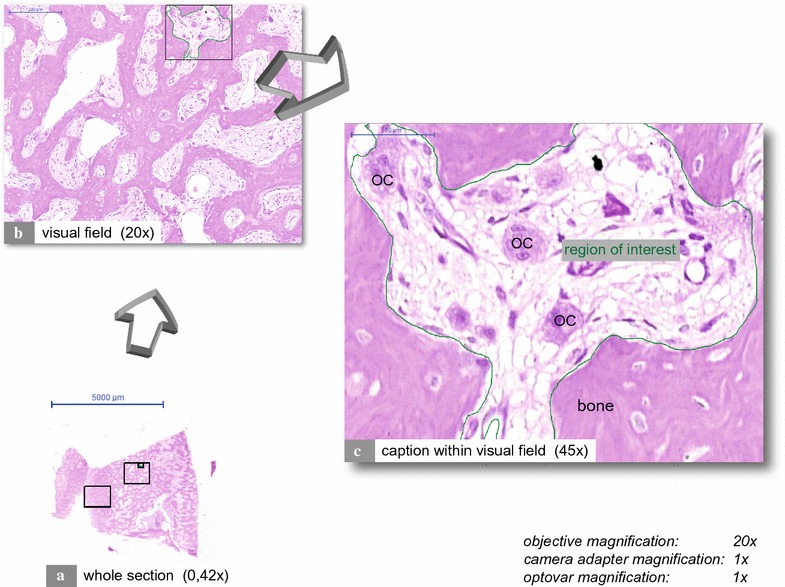
Whole slide imaging. The figure depicts an H&E stained MRONJ (BP) section consisting of mainly vital jaw bone. The histological section was scanned using a Pannoramic 250 Flash II Scanner (3DHISTECH Kft., Budapest, Hungary). Sections were analyzed via virtual microscopy using Pannoramic Viewer (3DHISTECH Kft., Budapest, Hungary). Within a scanned section, two visual fields were defined (**a**, *rectangles*). **b** Depicts a visual field. **c** Illustrates a caption within a visual field. Regions of interest (ROI) were marked, and their areas were calculated. Osteoclasts are tagged with “OC”

The “whole slide imaging” method was used for specimen analysis (see Fig. 1). All sections were scanned and digitalized completely after they had been quality-checked under a bright-field microscope (Axioskop, Zeiss, Jena, Germany; at a magnification of 100× to 400×). Scanning was performed in cooperation with the Institute of Pathology of the University Hospital Erlangen using a Pannoramic 250 Flash II Scanner (3DHISTECH Kft., Budapest, Hungary). All sections were analyzed via virtual microscopy using Pannoramic Viewer (3DHISTECH Kft., Budapest, Hungary) (Fig. 1). Two visual fields per section were set within the histological areas of interest with a high probability for the presence of osteoclasts. Such areas were subperiosteal bone, bone trabeculae, endosteal structures and connective tissue directly adjacent to the bone. If the visual field size exceeded the section size, only one visual field was used. Necrotic areas have been avoided because they are not appropriate for a meaningful histological analysis. Within a visual field, non-bony medullary tissues were defined as regions of interest (ROIs) (Fig. 1c). Any cell counting or analysis was performed only within ROIs. ROIs furthermore represented the medullary area. Cells had to meet at least the following criteria to be considered osteoclasts: (I) multinuclearity (at least two nuclei); (II) large cell body (larger than two fused mononuclear cells); (III) direct contact with bone or proximity to bone; and (IV) no proximity to granulomatous foci or foreign particles. Osteoclast morphology analysis was performed with Pannoramic Viewer, whereas quantitative analysis (cell counting) was performed with ImageJ (Rasband WS, ImageJ, US National Institutes of Health, Bethesda, MD, USA, http://imagej.nih.gov/ij/, 1997–2014). Quantitative analysis was performed by two medical students who were familiar with tissue morphology, IHC-methods and analysis. These students were blinded to the origin of the specimens. Inter-individual differences regarding cell counting were checked and did not exceed 10%.

### Statistics

For the morphology analysis, several osteoclasts within a visual field were analyzed for osteoclast diameter and nuclearity. The measurements made within a visual field were pooled, and the average for each osteoclast property was calculated accordingly.

For quantitative analysis the ratios of cells and osteoclasts to ROI were determined, and the osteoclast labeling indices (positive osteoclasts of a ROI/all osteoclasts of a ROI) were also determined.

The results are expressed as the minimum, maximum, average, median, interquartile range (IQR) and standard deviation (SD). Box plot diagrams visualize the respective values.

Statistical analysis was performed after consultation with the Department of Medical Informatics, Biometry and Epidemiology (IMBE) of the Friedrich-Alexander University Erlangen-Nürnberg. The Kolmogorov–Smirnov test was used to test for normal distribution. The Mann–Whitney U test was used for statistical hypothesis testing. p values ≤0.05 were considered statistically significant. SPSS (SPSS 24, IBM, New York, USA) was used.

## Results

### Differences in osteoclast number and morphology: many giant, hypernucleated osteoclasts in MRONJ (BP) specimens

**Fig. 2:**
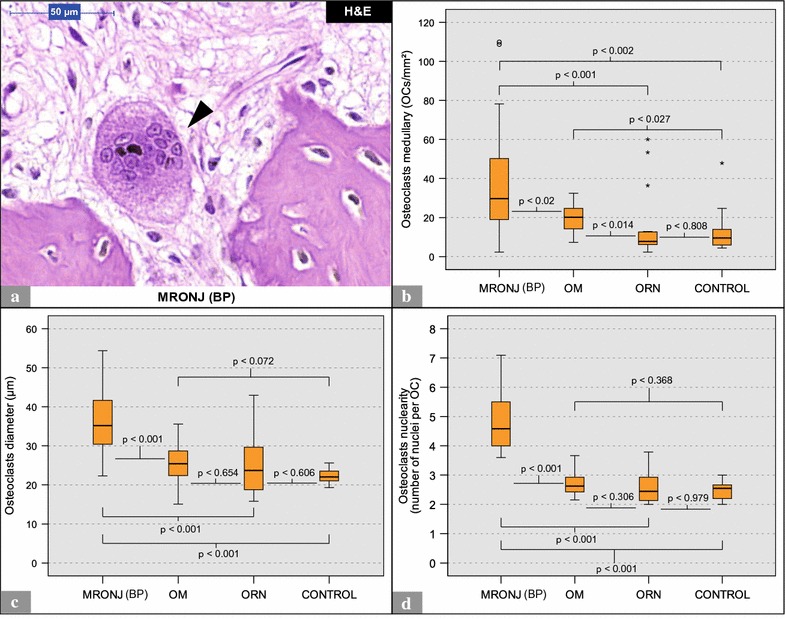
Quantitative and morphological analysis of osteoclasts (H&E): MRONJ (BP) vs. OM vs. ORN vs. control. **a** Depicts a giant hypernucleated osteoclast (*arrowhead*) within a MRONJ (BP) histological section. Note the pyknotic nuclei, the *round* shape, the detachment from the bone surface and the lack of ruffled borders. **b** Illustrates the number of osteoclasts per ROI. **c**, **d** Illustrate morphological differences in osteoclasts. * and ° mark statistical outliers. For detailed data see Table 2. *MRONJ* (*BP*) medication-related osteonecrosis of the jaw secondary to bisphosphonate therapy, *OC* osteoclasts, *OM* osteomyelitis, *ORN* osteoradionecrosis, *DC-STAMP* dendritic cell-specific transmembrane protein, *TRAP* tartrate-resistant acid phosphatase

**Fig. 3:**
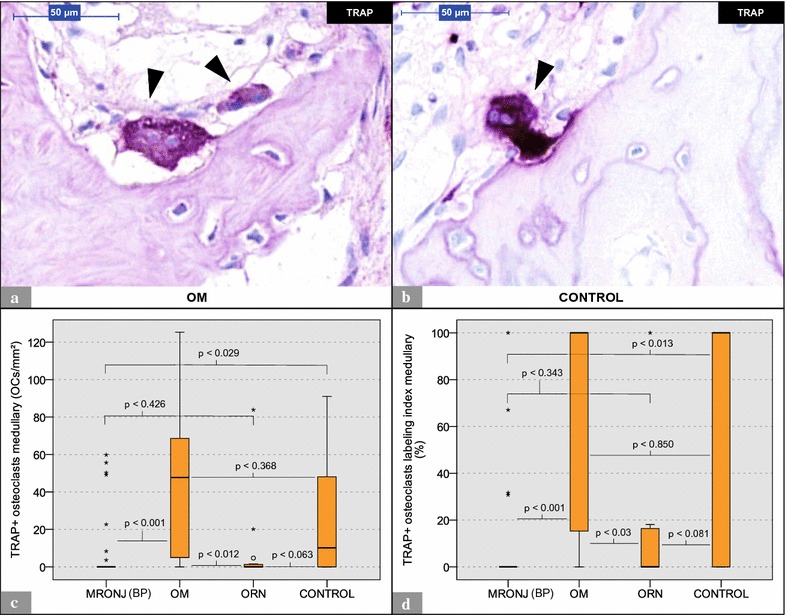
Analysis of TRAP staining: MRONJ (BP) vs. OM vs. ORN vs. control. **a** Shows 2 TRAP+ osteoclasts (*arrowheads*) within an OM histological section. **b** Depicts a TRAP+ osteoclast (*arrowhead*) within a control histological section. Note the direct contact with the bone surface. **c** Illustrates the number of TRAP+ osteoclasts per ROI. **d** Depicts the respective osteoclast labeling indices. * marks statistical outliers. For detailed data see Table 2. *MRONJ* (*BP*) medication-related osteonecrosis of the jaw secondary to bisphosphonate therapy, *OC* osteoclasts, *OM* osteomyelitis, *ORN* osteoradionecrosis, *DC-STAMP* dendritic cell-specific transmembrane protein, *TRAP* tartrate-resistant acid phosphatase

**Fig. 4:**
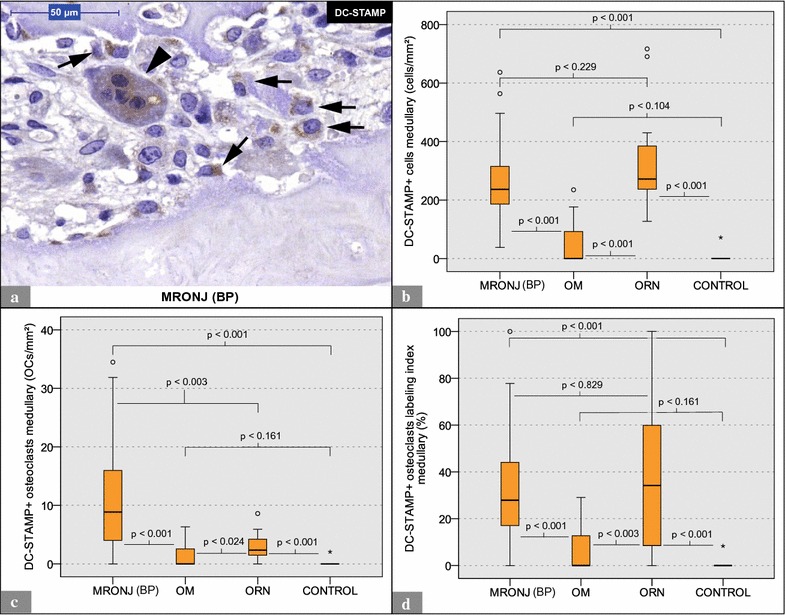
Analysis of (anti-)DC-STAMP staining: MRONJ (BP) vs. OM vs. ORN vs. control. **a** Shows a DC-STAMP+ osteoclast (*arrowhead*) and DC-STAMP+ mononuclear cells (*arrows*) within a MRONJ (BP) histological section. **b** Illustrates the number of DC-STAMP+ cells per ROI. **c** Depicts the number of DC-STAMP+ osteoclasts per ROI. **d** Illustrates the respective osteoclast labeling indices. * and ° mark statistical outliers. For detailed data, see Table 2. *MRONJ* (*BP*) medication-related osteonecrosis of the jaw secondary to bisphosphonate therapy, *OC* osteoclasts, *OM* osteomyelitis, *ORN* osteoradionecrosis, *DC-STAMP* dendritic cell-specific transmembrane protein, *TRAP* tartrate-resistant acid phosphatase

The quantitative analysis revealed the highest numbers of osteoclasts per ROI to be present in MRONJ (BP) specimens (Table 2; Fig. 2b). OM specimens featured significantly higher numbers of osteoclasts per ROI than ORN and control specimens (Table 2; Fig. 2b). The morphological analysis revealed that osteoclasts in MRONJ (BP) specimens were significantly larger (diameter) (Table 2; Fig. 2c) and featured significantly more nuclei (Table 2; Fig. 2d) than those present in specimens from other groups. On average, MRONJ (BP) osteoclasts were 1.4 times bigger than OM osteoclasts, 1.4 times bigger than ORN osteoclasts and 1.6 times bigger than control osteoclasts (Table 2). On average, MRONJ (BP) osteoclasts had 1.8 times more nuclei than OM osteoclasts, 1.9 times more nuclei than ORN osteoclasts and 1.9 times more nuclei than control osteoclasts (Table 2). No significant differences in osteoclast cell size and nuclearity were found between OM, ORN and control specimens. Often, the nuclei of giant hypernucleated osteoclasts appeared to be pyknotic (Fig. 2a). Compared to the elongated-oval osteoclasts that were present in OM, ORN and control specimens (e.g., Figs. 3a, b, 4a), osteoclasts in MRONJ (BP) specimens often featured round shapes (Fig. 2a). For detailed data, see Table 2. For p values, see Fig. 2.

**Table 2:** Descriptive data

	Group	Min	Max	Average	Median	IQR	SD
Hematoxylin and eosin (H&E) staining							
Diameter of osteoclasts (µm)	MRONJ (BP)	22.3	54.4	35.7	35.2	11.3	7.6
	OM	15.1	35.6	25.5	25.4	7.2	5.4
	ORN	15.8	43.0	25.3	23.7	12.7	8.1
	CONTROL	19.3	25.6	22.3	22.0	3.1	2.0
Nuclearity of osteoclasts (nuclei/osteoclast)	MRONJ (BP)	3.6	7.1	4.8	4.6	1.5	1.0
	OM	2.2	3.7	2.7	2.6	0.7	0.4
	ORN	2.0	3.8	2.6	2.4	0.9	0.6
	CONTROL	2.0	3.0	2.5	2.6	0.5	0.3
Osteoclasts per ROI (osteoclasts/mm^2^)	MRONJ (BP)	2.3	110.0	38.9	29.7	32.6	27.7
	OM	7.3	32.5	20.0	20.2	12.0	7.3
	ORN	2.3	60.1	16.0	7.8	6.9	18.4
	CONTROL	4.4	47.9	14.0	9.5	10.8	13.2
TRAP staining							
TRAP+ osteoclasts per ROI (osteoclasts/mm^2^)	MRONJ (BP)	0.0	59.8	8.3	0.0	0.9	18.7
	OM	0.0	125.4	44.3	47.7	69.6	39.0
	ORN	0.0	83.9	7.5	0.0	1.5	21.8
	CONTROL	0.0	91.1	25.6	10.1	53.5	32.6
Labeling index (%/100)	MRONJ (BP)	0	1	0.176	0	0.077	0.357
	OM	0	1	0.687	1	1	0.464
	ORN	0	1	0.231	0	0.180	0.403
	CONTROL	0	1	0.686	1	1	0.476
Anti-DC-STAMP staining							
Positive cells per ROI (cells/mm^2^)	MRONJ (BP)	38.3	636.8	266.6	236.6	146.9	147.2
	OM	0.0	235.1	61.1	0.0	102.9	79.8
	ORN	127.1	716.7	327.7	272.1	187.3	174.3
	CONTROL	0.0	71.8	7.2	0.0	0.0	22.7
DC-STAMP+ osteoclasts per ROI (osteoclasts/mm^2^)	MRONJ (BP)	0.0	34.5	10.9	8.9	12.4	9.6
	OM	0.0	6.3	1.3	0.0	2.6	1.9
	ORN	0.0	8.6	3.1	2.3	3.6	2.3
	CONTROL	0.0	2.1	0.2	0.0	0.0	0.6
Labeling index (%/100)	MRONJ (BP)	0	1	0.331	0.279	0.290	0.254
	OM	0	0.290	0.066	0	0.150	0.093
	ORN	0	1	0.375	0.342	0.650	0.333
	CONTROL	0	0.083	0.008	0	0	0.026
Analysis within MRONJ (BP) group							
Diameter of osteoclasts (µm)	DC-STAMP+ OCs	13.8	29.2	22.3	22.5	6.4	4.0
	Average OCs (H&E)	22.3	54.4	35.7	35.2	11.3	7.6
Nuclearity of osteoclasts (nuclei/osteoclast)	DC-STAMP+ OCs	2.0	3.4	2.5	2.5	0.4	0.4
	Average OCs (H&E)	3.6	7.01	4.8	4.6	1.0	1.0

Min, minimum; Max, maximum; IQR, interquartile range; SD, standard deviation; H&E, hematoxylin and eosin stain; MRONJ (BP), medication-related osteonecrosis of the jaw secondary to bisphosphonate therapy; OM, osteomyelitis; ORN, osteoradionecrosis; DC-STAMP, dendritic cell-specific transmembrane protein; TRAP, tartrate-resistant acid phosphatase; MRONJ (BP), medication-related osteonecrosis of the jaw secondary to bisphosphonate therapy; OC, osteoclasts; OM, osteomyelitis; ORN, osteoradionecrosis; DC-STAMP, dendritic cell-specific transmembrane protein; TRAP, tartrate-resistant acid phosphatase

## Osteoclastic TRAP expression: low expression in MRONJ (BP) and ORN specimens

TRAP-positive (TRAP+) cells showed a red coloration of the whole cell body (Fig. 3a, b). TRAP-positivity was mainly observed in osteoclasts but also in a few scattered non-osteoclastic cells. TRAP+ osteoclasts were found in specimens from all groups. However, the amount of TRAP+ osteoclasts per ROI were found to be significantly smaller in MRONJ (BP) and ORN specimens than in OM and control specimens (no significant difference for ORN vs. control) (Table 2; Fig. 3c). Furthermore, the osteoclast labeling indices for TRAP were significantly lower in MRONJ (BP) and ORN specimens than in OM and control specimens (Table 2; Fig. 3d; no significant difference for ORN vs. CONTROL). For detailed data, see Table 2. For p values, see Fig. 3.

## High DC-STAMP expression in MRONJ (BP) and ORN specimens

**Fig. 5:**
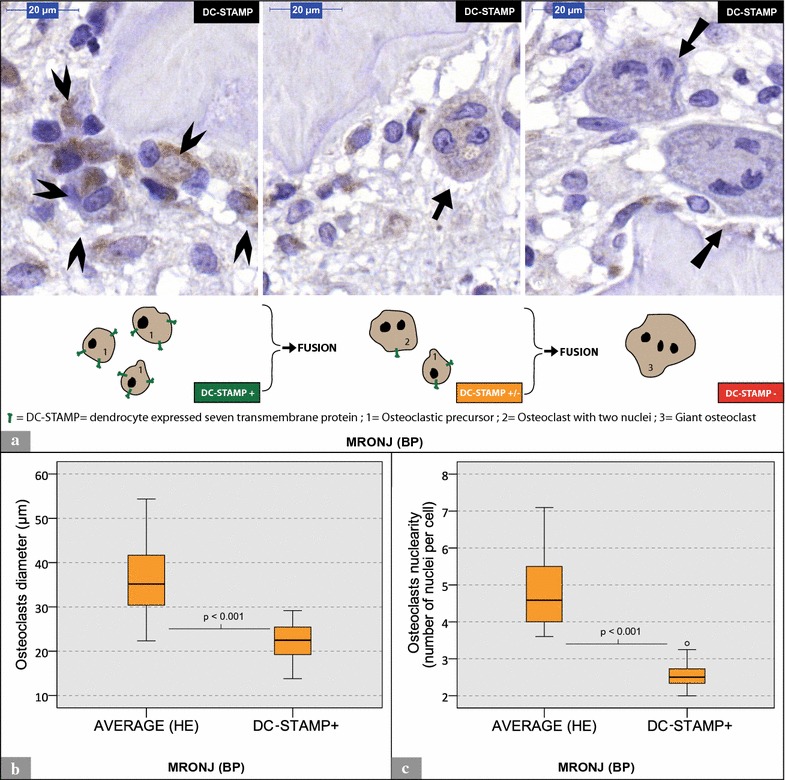
Analysis of OCs within MRONJ (BP) specimens. **a** Shows three different visual fields of the same MRONJ (BP) histological section [(anti-)DC-STAMP staining]. Nuclear counterstaining was performed. *Arrowheads* mark a bundle of potential osteoclastic precursors with a high expression of DC-STAMP. The *arrow* marks an osteoclast with intermediate DC-STAMP expression. *Double arrows* tag giant hypernucleated osteoclasts with no DC-STAMP expression. Below the visual fields, an illustration model depicts the gradual loss of DC-STAMP receptors during osteoclastogenesis. **b**, **c** Show the comparison between DC-STAMP+ osteoclasts and average osteoclasts (H&E). **b** Illustrates differences in osteoclast diameter. **b** Visualizes differences in number of nuclei per osteoclast

DC-STAMP-positive (DC-STAMP+) cells showed a brown membranous and cytoplasmic coloration (Figs. 4a, 5a). DC-STAMP+ osteoclasts and DC-STAMP+ mononuclear cells were found in specimens from all groups. The quantitative analysis revealed a significantly higher ratio of DC-STAMP+ cells (mononucleated cells and osteoclasts) per ROI in MRONJ (BP) and ORN specimens than in OM and control specimens (Table 2; Fig. 4b). MRONJ (BP) and ORN specimens featured significantly more DC-STAMP+ osteoclasts per ROI than OM and control specimens (Table 2; Fig. 4c). Furthermore, the osteoclast labeling indices were significantly higher in MRONJ (BP) and ORN specimens than in OM and control specimens (Table 2; Fig. 4d). For detailed data, see Table 2. For p values, see Fig. 4.

## MRONJ (BP) specimens: DC-STAMP+ osteoclasts vs. average osteoclasts (H&E)

Giant hypernucleated osteoclasts tended to be DC-STAMP-negative (DC-STAMP) (Fig. 5a). The comparison between DC-STAMP staining and H&E staining for each MRONJ (BP) specimen revealed that DC-STAMP+ osteoclasts were significantly smaller (p < 0.001; Fig. 5b) and had significantly fewer nuclei (p < 0.001; Fig. 5c) than the collective's average OCs (H&E). For detailed data, see Table 2.

## Discussion

### Anomalies of osteoclasts in MRONJ (BP) specimens

Due to the osteoclast-modulating effects of bisphosphonates, osteoclast morphology has long been the focus of histological investigations of MRONJ (BP) tissues.

High numbers of osteoclasts were observed in jaw bone specimens from patients with osteonecrosis after pamidronate and zoledronate treatment [44]. However, a depletion of osteoclasts in animal MRONJ (BP) tissue was described as well [32]. The present study demonstrated high numbers of osteoclasts in MRONJ (BP) tissues. MRONJ (BP) specimens featured the highest quantity of osteoclasts per ROI of all examined specimens. The effects of bisphosphonates on osteoclasts as well as the condition of the inflammation could influence the osteoclast quantity in MRONJ (BP) tissue.

The inconsistency of results in the literature with respect to the quantity of osteoclasts in MRONJ (BP) tissues indicates the relevance of an osteoclast analysis, which not only investigates osteoclast count but also osteoclast function. Several studies (and case reports) on animals [45–47] and patients with osteopetrosis [48], osteoporosis [21] and osteogenesis imperfecta [49] indicated that the administration of bisphosphonates is associated with the presence of giant, hypernucleated osteoclasts in bone tissues. These altered cells were also described in MRONJ (BP) tissues [44]. This finding is in line with the observations made in the present study.

The farnesyl pyrophosphate synthase and mevalonate pathway is fundamental for the osteoclast function and morphology [50, 51]. This pathway involves the production of small guanosine triphosphate—binding proteins, which are essential for the formation of ruffled borders [52].

The number of osteoclasts is assumed to be an index of bone resorption [53]. However, this assumption needs to be questioned for MRONJ (BP) because most of the many osteoclasts in MRONJ (BP) specimens observed in the present study were detached from the bone surface, had no ruffled borders and featured round cell shapes (e.g., Fig. 2a). These morphological features of osteoclasts were also described in animals and patients after bisphosphonate treatment [21, 54, 55]. The bisphosphonate-associated interference in the farnesyl and mevalonate pathway and the consecutive loss of small guanosine triphosphate—binding proteins might explain the detachment of osteoclasts from the bone surface.

Animal model studies indicated a bisphosphonate-associated inhibition of TRAP expression [56, 57]. Patients receiving ibandronate for bone metastases presented decreased serum TRAP-5b concentrations [58]. The present study also showed a low osteoclast expression of TRAP in MRONJ (BP) specimens. In addition to the observed detachment of osteoclasts from the bone surface, this finding indicates a functional disorder of osteoclasts in MRONJ (BP).

The present study, for the first time, analyzed DC-STAMP expression in human jaw bone specimens. The results show a high expression of DC-STAMP by osteoclasts and mononuclear cells (potential osteoclastic precursors), indicating a high rate of cell–cell-fusion in MRONJ (BP) tissues.

There appears to be no direct positive correlation between cell–cell fusion (DC-STAMP) and the resorptive activity (TRAP) of osteoclasts in MRONJ (BP) because DC-STAMP expression was high and TRAP expression was low in MRONJ (BP) specimens.

The examination of DC-STAMP+ osteoclasts in MRONJ (BP) specimens surprisingly revealed that DC-STAMP+ osteoclasts were significantly smaller and featured fewer nuclei than average MRONJ (BP) osteoclasts, which had been analyzed in H&E staining. In vitro, it was observed that the gradual increase of osteoclast size and nuclearity directly contrasts with the decline of osteoclast DC-STAMP expression [31]. This finding could be explained by gradual internalization of DC-STAMP from the cell membrane to the cytoplasm during osteoclastogenesis [33]. This internalization might also explain the abovementioned membranous and cytoplasmic expression of osteoclasts after IHC-staining with anti-DC-STAMP-antibodies.

### What does the comparison with OM tell us?

The distinction between acute, subacute and chronic OM was not addressed in the present study because the focus was only on the microbial cause of this condition (no bisphosphonates, no radiation) [15, 16]. Patients with the diagnosis of primary chronic OM (non-bacterial cause) were not included in this study. Because OM of the jaw is considered to have a polymicrobial origin, the used bone samples were not examined for an OM-triggering microbe [14].

The numbers of osteoclasts per ROI in OM specimens were significantly higher than in ORN and control specimens but significantly lower than in MRONJ (BP) specimens. Osteoclast TRAP expression was high and DC-STAMP expression was low in osteomyelitis specimens. The elevated numbers of osteoclasts together with a high TRAP expression indicate a high resorptive activity of osteoclasts in osteomyelitis specimens. This finding might be triggered by bacteria or bacterial products, as these were demonstrated to induce osteoclastogenesis and osteoblast RANKL production, which might also stimulate osteoclast activity [59–61].

The comparison between OM and MRONJ (BP) revealed two contrary osteoclast profiles regarding TRAP and DC-STAMP expression. This result might indicate different pathogenic mechanisms. The activation of osteoclasts in OM might be part of a reactive response to a microbial infection, whereas the functional disorder of osteoclasts by bisphosphonates could be the cause of MRONJ (BP).

### What does the comparison with ORN tell us?

In vitro, it was shown that radiation inhibits osteoclastic progenitor cells and therefore disrupts osteoclast formation [62]. However, it was also demonstrated in vivo that radiation exposure might elicit a pro-resorptive state that is associated with high numbers of osteoclasts [63]. Comparative osteoclast counts in jaw bone sections of ORN and MRONJ (BP) patients have already been described in literature [64]. In accordance with the data of the present study, they found lower numbers of osteoclasts in ORN specimens than in MRONJ (BP) specimens (Fig. 2b) [64]. However, contrary to the present study, they demonstrated significantly higher numbers of osteoclasts in both types of osteonecrosis when compared with a control group [64]. In accordance with the mentioned literature we found ORN lesions to feature homogenous necrosis extension patterns, whereas MRONJ (BP) lesions showed residual nests of vital bone [44, 64]. These histological differences could influence the meaningfulness of comparative cell counts.

Similarities between osteoclasts in ORN and MRONJ (BP) specimens seem to exist. The present study demonstrated a low osteoclastic TRAP expression and a high expression of DC-STAMP for both diseases (Figs. 3, 4). Therefore, the inhibition of osteoclast activity and the acceleration of cell–cell fusion appear to play a role in the pathogenesis of both types of osteonecrosis. However, giant hypernucleated osteoclasts were found only in MRONJ (BP) specimens. This result indicates that the presence of these altered cells in MRONJ (BP) tissue could not be explained by the mechanism of cell–cell fusion alone because a high level of DC-STAMP expression was detected in MRONJ (BP) and ORN tissues. Furthermore, this finding is stressed by our observation that the DC-STAMP expression of large osteoclasts is usually low (Fig. 5). At this point, the bisphosphonate-mediated prevention of apoptosis must also be considered [21]. The presence of these abnormal cells in MRONJ (BP) tissues might be due to accelerated cell–cell-fusion and the prevention of apoptosis. Giant hypernucleated osteoclasts might not occur in ORN tissues, because, although the cell–cell-fusion rate is high, the bisphosphonate-mediated prevention of apoptosis is not present. Further studies of osteoclastic regulators are necessary to fully understand the genesis of giant, hypernucleated osteoclasts in MRONJ (BP).

### From bench-to-beside?

Bisphosphonates, radiation and microbes are agents that all could cause destruction of the human jaw bone. The present study illustrated the differences in osteoclast profiles of these common osteopathologies of the human jaw. Although these osteoclast profiles could not yet be assumed to be disease-specific, they could be considered characteristic of the respective diseases. However, they might facilitate the histopathological differentiation between MRONJ (BP), ORN and OM. This differentiation is of clinical interest because the therapeutic approaches for these diseases differ.

### Limitations

Among the analyzed diseases, only MRONJ (BP) features giant hypernucleated osteoclasts. However, these cells have also been observed in bone specimens from patients with Schönberg disease and osteopetrosis type II [65]. The term MRONJ includes osteonecrosis of the jaw related to the treatment with other anti-resorptive (e.g., denosumab) and anti-angiogenic medications [10, 66]. Histological comparisons between MRONJ (BP) and the mentioned diseases are not part of the present study but need to occur in future studies.

The analysis of patient samples is associated with the problem that some factors (e.g., age, lifestyle, nutrition), possibly influencing the jaw bone homeostasis, might remain unconsidered.

This study does not consider standardized collectives, sample sizes or localization within the jaw, because the specimens were derived from routine biopsies.

This study does not include the analysis of bisphosphonate-affected jaw bone without necrosis.

## Conclusion

This study indicates that the osteoclast profile of MRONJ (BP) is characterized by osteoclast inactivation (low secretion/expression of TRAP) and a high cell–cell fusion rate (high expression of DC-STAMP). For the first time, the present study analyzed the expression of DC-STAMP in routine human jaw bone specimens. We found similar expression patterns for DC-STAMP in MRONJ (BP) and ORN specimens, although giant, hypernucleated osteoclasts were found in MRONJ (BP) specimens only. This finding indicates that the emergence of these altered osteoclasts in MRONJ (BP) cannot be attributed to an increased DC-STAMP-triggered cell–cell fusion alone.

The incidental characterization of the osteoclast profiles of OM and ORN revealed striking differences, that might facilitate the histopathological differentiation between MRONJ (BP), ORN and OM. An accurate differentiation is essential because therapeutic approaches for these diseases are somewhat different.

## Abbreviations

DC-STAMP: dendritic cell-specific transmembrane protein
H&E: hematoxylin and eosin staining
MRONJ (BP): medication-related osteonecrosis of the jaw secondary to bisphosphonate therapy
OM: osteomyelitis
ONJ: osteonecrosis of the jaw
ORN: osteoradionecrosis
RANK: receptor activator of nuclear factor κ-B
RANKL: receptor activator of nuclear factor κ-B ligand
ROI: region of interest
TRAP: tartrate-resistant acid phosphatase
